# Cloning and expression of a zebrafish *SCN1B *ortholog and identification of a species-specific splice variant

**DOI:** 10.1186/1471-2164-8-226

**Published:** 2007-07-10

**Authors:** Amanda J Fein, Laurence S Meadows, Chunling Chen, Emily A Slat, Lori L Isom

**Affiliations:** 1 Department of Pharmacology, University of Michigan, Ann Arbor, MI 48109-0632, USA

## Abstract

**Background:**

Voltage-gated Na^+ ^channel β1 (*Scn1b*) subunits are multi-functional proteins that play roles in current modulation, channel cell surface expression, cell adhesion, cell migration, and neurite outgrowth. We have shown previously that β1 modulates electrical excitability *in vivo *using a mouse model. *Scn1b *null mice exhibit spontaneous seizures and ataxia, slowed action potential conduction, decreased numbers of nodes of Ranvier in myelinated axons, alterations in nodal architecture, and differences in Na^+ ^channel α subunit localization. The early death of these mice at postnatal day 19, however, make them a challenging model system to study. As a first step toward development of an alternative model to investigate the physiological roles of β1 subunits *in vivo *we cloned two β1-like subunit cDNAs from *D. rerio*.

**Results:**

Two β1-like subunit mRNAs from zebrafish, *scn1ba_tv1 *and *scn1ba_tv2*, arise from alternative splicing of *scn1ba*. The deduced amino acid sequences of Scn1ba_tv1 and Scn1ba_tv2 are identical except for their C-terminal domains. The C-terminus of Scn1ba_tv1 contains a tyrosine residue similar to that found to be critical for ankyrin association and Na^+ ^channel modulation in mammalian β1. In contrast, Scn1ba_tv2 contains a unique, species-specific C-terminal domain that does not contain a tyrosine. Immunohistochemical analysis shows that, while the expression patterns of Scn1ba_tv1 and Scn1ba_tv2 overlap in some areas of the brain, retina, spinal cord, and skeletal muscle, only Scn1ba_tv1 is expressed in optic nerve where its staining pattern suggests nodal expression. Both *scn1ba *splice forms modulate Na^+ ^currents expressed by zebrafish *scn8aa*, resulting in shifts in channel gating mode, increased current amplitude, negative shifts in the voltage dependence of current activation and inactivation, and increases in the rate of recovery from inactivation, similar to the function of mammalian β1 subunits. In contrast to mammalian β1, however, neither zebrafish subunit produces a complete shift to the fast gating mode and neither subunit produces complete channel inactivation or recovery from inactivation.

**Conclusion:**

These data add to our understanding of structure-function relationships in Na^+ ^channel β1 subunits and establish zebrafish as an ideal system in which to determine the contribution of *scn1ba *to electrical excitability *in vivo*.

## Background

Voltage gated Na^+ ^channel β1 (*Scn1b*) subunits are multi-functional proteins that participate in inter- and intra-cellular communication on multiple time scales via modulation of electrical signal transduction and cell adhesion [1,2]. β1 subunits modulate Na^+ ^currents [3], regulate the level of Na^+ ^channel cell surface expression [4], and participate in cell adhesive interactions that lead to changes in cell migration [5], cellular aggregation [6], cytoskeletal recruitment [7,8], and/or neurite outgrowth *in vitro *[9]. Mice lacking β1 subunits exhibit seizure activity, ataxia, slowed action potential conduction, decreased numbers of mature nodes of Ranvier in myelinated axons, alterations in nodal architecture, and differences in Na^+ ^channel α subunit localization [10]. Thus, β1 subunits play critical roles in electrical excitability *in vivo*. However, while *Scn1b *null mice are interesting, their early death at postnatal day 19 and complex phenotype make them a challenging model system.

As a first step toward development of an alternative model system in which to study the physiological roles of Na^+ ^channel β1 subunits *in vivo *we chose *D. rerio*. This is an attractive model system with a number of advantages over mice, including the production of large numbers of embryos per single pair mating, external fertilization with transparent larvae allowing for genetic manipulation from the one cell stage, and rapid development [11]. Embryos contain most of their adult structures by 48 hours post-fertilization (hpf) and the majority of external and internal organs reach maturity by 5 days post-fertilization (dpf). Other commonly studied genetic model systems such as *Drosophila *or *C. elegans *were not appropriate for an *in vivo *investigation of Na^+ ^channel β subunits. There are no obvious candidates for voltage-gated Na^+ ^channel gene orthologs in the genome of *C. elegans *[12]. While the *Drosophila *genome encodes two Na^+ ^channel α subunit genes, orthologs of Na^+ ^channel β subunits appear to be lacking, suggesting that these subunits arose in evolution after the appearance of invertebrates [13]. Na^+ ^channel α subunit genes have been extensively studied in zebrafish, where eight different *SCNA *orthologs have been identified [14,15]. This is the first description of the structure and localization of a zebrafish Na^+ ^channel β subunit ortholog, although sequences of *SCN2B*, *SCN3B*, and *SCN4B *orthologs have recently been reported in GenBank. In the present study we report the cloning and expression of zebrafish *scn1ba*. Two alternate splice forms of *scn1ba *with distinct C-terminal domains, *scn1ba_tv1 *and *scn1ba_tv2*, are expressed in zebrafish mRNA. Both modulate Na^+ ^currents expressed by zebrafish *scn8aa *α subunits. *In situ *hybridization and immunohistochemical experiments show localization of zebrafish β1 subunits in brain, spinal cord, sensory neurons, and skeletal muscle. Interestingly, one of the splice variants, Scn1ba_tv1, is expressed in optic nerve while the other splice variant, Scn1ba_tv2, is not detectable in this tissue. Zebrafish are an ideal system in which to determine the contribution of Na^+ ^channel β1 subunits to neuronal development and to the establishment and maintenance of electrical excitability *in vivo*.

## Results and Discussion

### Zebrafish scn1ba is expressed as two splice variants

The Sanger zebrafish database was searched for translated ESTs with homology to the rat Scn1b peptide sequence (GenBank AAH94523), which shares 96% to 99% identity with the mouse, rabbit, and human β1 homologs [16]. Short regions of homology were identified and several ESTs were aligned. Portions of these sequences were used to design forward and reverse oligonucleotide PCR primers that were then used to amplify a 102 base pair product from a zebrafish retinal library as described in *Methods*. This product encoded a cDNA with a predicted peptide sequence containing high homology to rat Scn1b. We then performed RACE followed by an additional round of PCR using nested primers to generate a full-length cDNA. This reaction resulted in the amplification of two clones, each encoding peptides with significant homology to Scn1b: *scn1ba_tv1 *and *scn1ba_tv2*, respectively.

**Figure 1:**
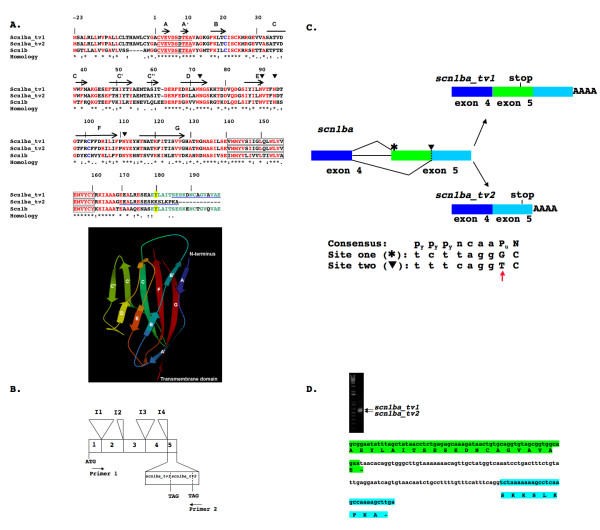
**Zebrafish *scn1ba_tv1 and scn1ba_tv2 are *splice variants**. **A**. Upper panel: Comparison of Scn1ba_tv1, Scn1ba_tv2 and Scn1b amino acid sequences. Amino acid residues that are identical are indicted in red, strongly similar substitutions are indicated by (:), and weakly similar amino acids are indicated by (.). Identical resides in exon 5 of Scn1ba_tv1 and Scn1b are indicated in green. The two cysteine residues predicted to form the Ig loop are indicated in blue. The conserved regions that form the A/A' face of the Ig loop, sites of interaction with theα subunit [17], are underlined. Tyrosine-181 in Scn1b and the corresponding residues in Scn1ba_tv1 and Scn1ba_tv2 are highlighted in yellow. Predicted sites of N-linked glycosylation are indicated by ▼. These sites were determined using NetNGlyc 1.0 [61]. Transmembrane segments are indicated as boxes. Peptides used for antibody generation are underlined in blue. Predicted β-sheets in the Ig loop domain, based on the crystal structure of myelin P_0 _[62], are shown with labeled arrows and correspond to the ribbon diagram included in the lower panel. Lower panel: Proposed three-dimensional structure of the Ig domain of β1 using the crystal structure of myelin Po (PDB 1NEU) as a template. The figure was created with the KiNG Viewer program via the RCSB Protein Data Bank web site [63]. β strands corresponding to the arrows in the upper panel are labeled A through G. **B**. Schematic showing the genomic organization of zebrafish *scn1ba*. The positions of introns 1 through 5 (I1 – I5) are indicated. Positions of primers used for RT-PCR in panel D are indicated. The C-terminal alternate splice domains contained in *scn1ba_tv1 *and *scn1ba_tv2 *are encoded by exon 5. **C**. Model of alternative splicing of *scn1ba*. Exons 4 and 5 (boxes) and intron 4 (line) are illustrated. The splice acceptor sequence at the beginning of exon 5 is indicated by  and the internal alternate splice acceptor site in exon 5 is indicated by a dashed line and by ▼. The location of stop codons in the resulting mRNAs are indicated. Drawings are not to scale. Consensus splice acceptor sequence [22] and the acceptor sequences found in exon 5 are indicated in the lower portion of the panel. P_y_: pyrimidine. P_u_: purine. Lower case: intronic sequence. Upper case: exonic sequence. The "T" indicated by the red arrow in the internal, alternate acceptor is rare and significantly weakens the site [22]. **D**. RT-PCR from whole fish RNA demonstrating that both splice variants of *scn1ba *are expressed in the mRNA pool. The upper band corresponds to *scn1ba_tv1 *and the lower band corresponds to *scn1ba_tv2*. Translations of the resulting alternate C-terminal splice products are shown below. The sequence highlighted in green is found in Scn1ba_tv1 and corresponds to the green portion of exon 5 illustrated in panel C. The sequence highlighted in turquoise is found in Scn1ba_tv2 and corresponds to the turquoise portion of exon 5 illustrated in panel C.

Alignment of the Scn1b, Scn1ba_tv1, and Scn1ba_tv2 peptide sequences is presented in Fig 1A. Overall, Scn1ba_tv1 and Scn1b share 53.15% identity, with 14.86% of residues strongly similar, and 12.16% of residues weakly similar. Zebrafish Scn1ba_tv2 shares 47.3% identity with Scn1b, with 13.96% of residues strongly similar, and 12.61% of residues weakly similar. Zebrafish Scn1ba_tv1 and Scn1ba_tv2 are identical except for their C-termini. Both zebrafish subunits contain a predicted N-terminal signal peptide followed by the start of the mature protein at residue alanine-1 (Fig. 1A) corresponding to the experimentally confirmed site in Scn1b [3]. Both subunits contain conserved cysteines predicted to form the extracellular β1 immunoglobulin (Ig) loop domain in mammals [17]. Residues forming the A/A' face of the Ig domain are conserved. This region has been shown to contain important sites of α-β1 interaction [17]. The zebrafish β subunits contain four predicted N-linked glycosylation sites. Three of the four correspond to those predicted in *Scn1b *(corresponding to the predicted zebrafish asparagine residues N-73, N-90, and N-94) [3]. The fourth site (zebrafish N-111), is not conserved in *Scn1b*. The fourth glycosylation site predicted in *Scn1b *corresponds to the predicted zebrafish residue histidine-114. Interestingly, this glycosylation site present in *Scn1b *is absent in *Scn3b *[18]. Comparing the Ig domain β sheets of Scn1ba_tv1 and Scn1ba_tv2 with Scn1b reveals significant differences in the C, C", F, and G strands, with the most significant differences in the C" region. The C-terminal domain of Scn1ba_tv2 is 12 amino acids shorter than that of Scn1ba_tv1 and lacks the C-terminal tyrosine corresponding to tyrosine-181 in Scn1b which has been demonstrated to be important for the recruitment of ankyrin, subcellular localization, and channel modulation [7,8]. Zebrafish Scn1ba_tv2 contains a lysine in the position corresponding to rat tyrosine-181, but within a novel C-terminal tail.

Zebrafish *scn1ba*, according to the zebrafish nomenclature convention [19], was identified on a BAC containing a contig from linkage group (LG) 16 (GenBank CR318611). Conserved synteny was found for the region of LG16 containing *scn1ba *and regions surrounding *SCN1B *on human chromosome 19 and *Scn1b *on mouse chromosome 7. Genes closely linked to *Scn1b *on the mouse chromosome were compared to this region of the zebrafish genome using the Blast program. Predicted genes for zebrafish orthologs were then mapped against the zebrafish Zv6 assembly [20] to determine their linkage group designations. Located in close apposition to *scn1ba *are *hepsin*, *gramd1a*, and *fxyd*, genes that are also closely linked with *Scn1b *in the mouse and *SCN1B *in human genomes, confirming that *scn1ba *is orthologous to the mammalian genes. Analysis of *scn1ba *showed exon-intron boundaries in agreement with the published sites for mammalian *SCN1B *[21]. The alternative C-terminal coding sequences contained in *scn1ba_tv1 *and *scn1ba_tv2 *were both found within exon 5 of *scn1ba*. Two alternative 3' splice acceptor sites, located at the beginning of exon 5 and internal to exon 5 respectively, separated by 97 base pairs, were identified (Fig. 1C). The internal acceptor site, initiating the *scn1ba_tv2 *C-terminus, is a weak, non-consensus sequence containing a rare thymidine as the first base of the internal exon (Fig. 1C, red arrow) [22]. To confirm the expression of each of these splice variants in the zebrafish mRNA pool, we performed a single RT-PCR reaction using whole fish RNA as template with a forward primer encoding the region corresponding to the A strand of the Ig domain shared by *scn1ba_tv1 *and *scn1ba_tv2 *and a reverse primer encoding the 3' end of the putative alternative C-terminal sequence of *scn1ba_tv2 *and found in the 3' untranslated region (UTR) of *scn1ba_tv1 *(Fig. 1B, "primer 1" and "primer 2", respectively). As shown in Fig. 1D, this reaction amplified two bands of 633 and 847 base pairs, respectively. These products were subsequently confirmed by DNA sequencing to be identical to the zebrafish *scn1ba_tv1 *and *scn1ba_tv2 *cDNAs cloned in the original reactions. Thus, *scn1ba *is expressed in zebrafish as two alternatively spliced products.

Mammalian *Scn1b *alternate splice products have been identified previously and this *Scn1b *alternate splicing appears to be species-specific. A *Scn1b *alternate splice product has been described in rat, β1.2, arising from retention of intron 5 and resulting in a novel 3' UTR [23]. Rat β1A [24] (corresponding to human β1B [25]) is encoded via retention of intron 3, generating β1 polypeptides with novel transmembrane and intracellular domains that are species-specific. Translation of *scn1ba *intron 3 in frame with exon 3 predicts a short peptide extension beyond exon 3 containing 11 amino acids (GRSIFTFIHFP) that would produce a truncated, soluble protein. We have not yet found evidence for expression of these alternatively spliced mRNAs in zebrafish.

### In Situ Hybridization analysis

**Figure 2:**
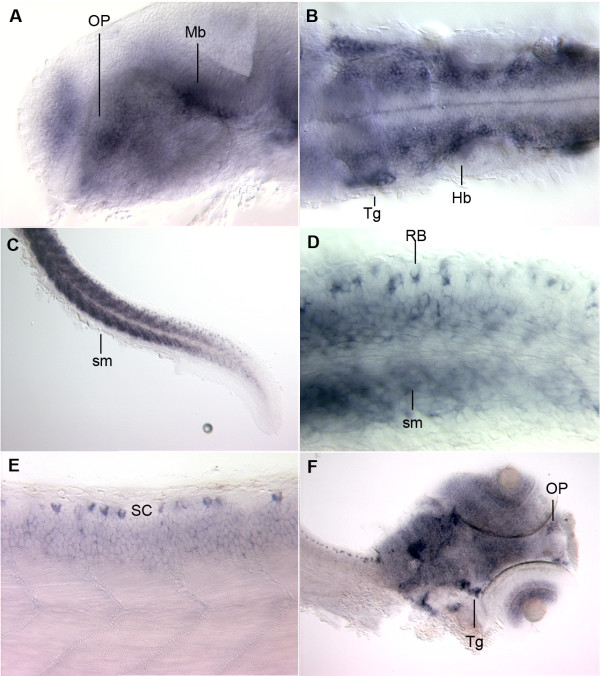
***In situ *hybridization**. **A – D**: Fish stained at 24 hpf. **A**. Staining is apparent in the olfactory placode (OP) and the midbrain (Mb). **B**. Dorsal mount showing staining in the trigeminal neuron (Tg) and in the rhombomeres of the hindbrain (Hb). **C**. Staining in spinal cord and skeletal muscle (sm). **D**. Higher magnification of Rohon Beard cells (RB) flanking skeletal muscle (sm). **E**. Fish at 48 hpf with staining in the Rohan Beard cells of the spinal cord (SC) and in the skeletal muscle. **F**. Staining throughout the brain, at the olfactory pits (OP), in the layers of the retina, and in the trigeminal ganglion (Tg) of fish at 72 hpf.

We used *in situ *hybridization analysis to investigate the expression of *scn1ba *mRNA at 24 hpf, 48 hpf, and 3 dpf. An anti-sense cRNA probe corresponding to both the 3' UTR and coding region of *scn1ba_tv1 *and *scn1ba_tv2 *was used. A sense probe was used as a control and resulted in no specific staining (data not shown). Because the alternative C-terminus expressed in *scn1ba_tv2 *is contained within the 3' UTR of *scn1ba_tv1*, these probes did not distinguish between the two subunits. Thus, these results represent the combined expression of the two mRNA species. As shown in Fig. 2A–D, we observed CNS expression beginning at 24 hpf in the olfactory placode (OP), midbrain (Mb) (Fig. 2A), hindbrain (Hb), and trigeminal neurons (Tg) (Fig. 2B). Extensive staining was also observed in the skeletal muscle cells (sm) of the trunk at 24 hpf (Fig. 2C). In addition to staining in skeletal muscle, staining was observed in the Rohon Beard neurons (RB) in the spinal cord (RB (Fig. 2D). At 48 hpf, expression patterns were not changed, although the overall intensity of staining in the brain had increased (data not shown). At 3 dpf (Fig. 2E) robust expression was visible in the spinal cord (SC), and expression was evident in the olfactory pit (OP) and the retina, as well as in the forebrain, midbrain, hindbrain and trigeminal ganglia (Tg) (Fig. 2F). Na^+ ^currents have been recorded in zebrafish Rohon Beard neurons *in vivo*. In these cells, Na^+ ^currents undergo developmentally regulated increases in amplitude, hyperpolarizing shifts in the voltage dependence of activation, and acceleration of fast inactivation [26,27]. These results are consistent with expression of a β1 subunit that increases with development, promoting cell surface channel expression, and modulating Na^+ ^current, and are supported by our *in situ *hybridization results.

### Antibody characterization

**Figure 3:**
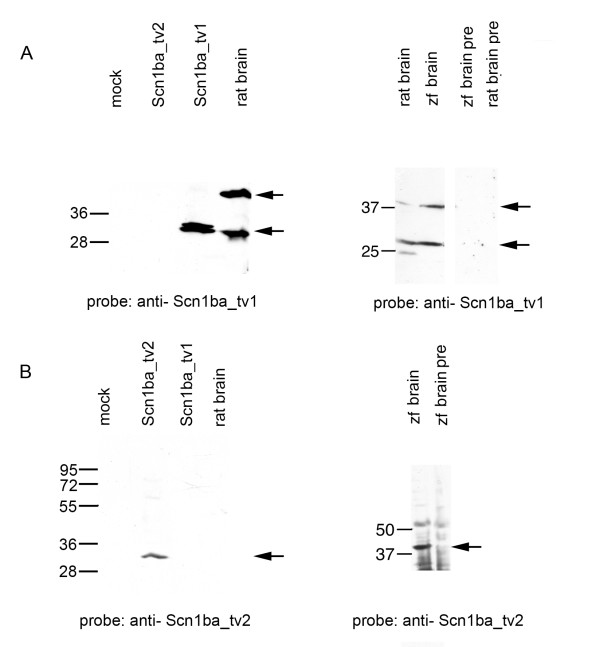
**Antibody characterization**. **A**. *Left panel: *Western blot probed with anti-Scn1ba_tv1. Lane 1: mock transfected Chinese hamster lung 1610 cells; Lane 2: Chinese hamster lung 1610 cells transiently transfected with *scn1ba_tv2 *cDNA; Lane 3: Chinese hamster lung 1610 cells transiently transfected with *scn1ba_tv1 *cDNA; Lane 4: 5 μg rat brain membranes. Arrows indicate immunoreactive bands at ~30 kD in the transfected cells and at ~30 kD and ~38 kD in rat brain. *Right panel: *Western blot probed with anti-Scn1ba_tv1. Lane 1: 5 μg rat brain membranes; Lane 2: 15 μg zebrafish brain membranes; Lane 3: 5 μg rat brain membranes probed with anti-Scn1ba_tv1 that had been preadsorbed to the immunizing peptide ("pre"); Lane 4: 15 μg zebrafish (zf) brain membranes probed with anti-Scn1ba_tv1 that had been preadsorbed to the immunizing peptide. Arrows indicate immunoreactive bands at ~30 kD and ~38 kD in both species. **B**. *Left panel: *Western blot probed with anti-Scn1ba_tv2. Lane 1: mock transfected Chinese hamster lung 1610 cells; Lane 2: Chinese hamster lung 1610 cells transiently transfected with *scn1ba_tv2 *cDNA; Lane 3: Chinese hamster lung 1610 cells transiently transfected with *scn1ba_tv1*; Lane 4: 5 μg rat brain membranes. Arrow indicates immunoreactive band at ~30 kD. *Right panel: *Western blot probed with anti-Scn1ba_tv2. Lane 1: 15 μg zebrafish brain membranes; Lane 2: 15 μg zebrafish brain membranes probed with anti-Scn1ba_tv2 that had been preadsorbed to the immunizing peptide ("pre"). Arrow shows immunoreactive band at ~38 kD.

To investigate whether both of the *scn1ba *slice products are expressed and if so, whether they are differentially localized in developing fish, polyclonal antibodies were designed to the unique C-terminal regions of each polypeptide. Fig. 3 demonstrates the specificity of these antibodies using Western blot analysis. Chinese hamster lung 1610 cells transiently transfected with cDNA encoding *scn1ba_tv1*, *scn1ba_tv2*, or empty vector ("mock") were tested, as well as rat brain and zebrafish brain membranes. In Fig. 3A, left panel, we show that anti-Scn1ba_tv1 recognized a protein band at ~30 kD in 1610 cells transfected with a *scn1ba_tv1 *expression plasmid, as well as two bands, at ~30 kD and ~38 kD, respectively, in rat brain. This result may reflect differential glycosylation of β1 in the cell line vs. brain, where both forms appear to be expressed. Protein bands were not detected in 1610 cells that were mock transfected or transfected with cDNA encoding *scn1ba_tv2*. Fig. 3A, right panel, shows anti-Scn1ba_tv1 immunoreactive bands at ~30 kD and ~38 kD in both rat brain and zebrafish brain. The immunoreactive signal was blocked following preadsorption of the antibody with its immunizing peptide. In a similar experiment, anti-Scn1ba_tv2 recognized a specific protein band at ~30 kD in 1610 cells transfected with a *scn1ba_tv2 *expression plasmid (Fig. 3B, left panel). No bands were detected in cells that were mock transfected or transfected with *scn1ba_tv1 *cDNA. In contrast to anti-Scn1ba_tv1, anti-Scn1ba_tv2 did not identify any protein bands in rat brain, consistent with our inability to identify a similar translated sequence from *Scn1b *exon 5, and suggesting that *scn1ba_tv2 *is a species-specific splice variant. In Fig. 3B, right panel, we show that anti-Scn1ba_tv2 recognizes a ~38 kD immunoreactive band in zebrafish brain that is blocked following preadsorption of the antibody with the immunizing peptide. Again, we propose that there are differences in the extent of β1 glycosylation in the cell line vs. brain.

The specificity of these antibodies was further demonstrated using immunohistochemical methods. Fluorescent signals from the anti-Scn1ba_tv1 or anti-Scn1ba_tv2 antibodies were blocked by pre-absorption of each antibody with its corresponding peptide for 1 h at room temperature [see Additional file 1]. Normally, anti-Scn1ba_tv1 and anti-Scn1ba_tv2 both show robust staining in the retina as shown below, however pre-incubation with the corresponding peptides dramatically reduced the signals to background levels.

### Zebrafish Scn1ba_tv1 and Scn1ba_tv2 protein expression

Anti-Scn1ba_tv1 and anti-Scn1ba_tv2 antibodies were used to determine the localization of these subunits in fish. We showed previously that a key tyrosine residue (tyrosine-181) in the C-terminus of Scn1b is critical for β1-ankyrin interactions and β1 subcellular localization [7,8,28]. Because the C-terminal domain of Scn1ba_tv1 contains a tyrosine residue in the position corresponding to tyrosine-181 in Scn1b while Scn1ba_tv2 does not, we hypothesized that these subunits may be differentially localized *in vivo*. Antibodies specific to each subunit were used to stain both whole fish and cryosectioned fish to test this hypothesis.

**Figure 4:**
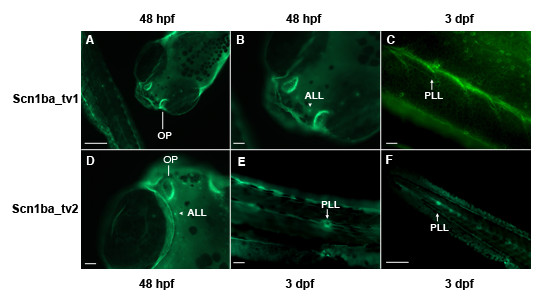
**Zebrafish Scn1ba_tv1 and Scn1ba_tv2 expression in early sensory systems**. Panels A – C: anti-Scn1ba_tv1. Panels D – F: anti-Scn1ba_tv2. **A**. 48 hpf fish showing staining in the olfactory pit (OP) and in neuromasts of the anterior (ALL) and posterior (PLL) lateral line systems. **B**. Higher magnification of head region from panel A showing the anterior lateral line (ALL). **C**. 3 dpf fish showing staining in the posterior lateral line (PLL) of the trunk. **D**. 48 hpf fish showing staining in olfactory pit (OP) and anterior lateral line (ALL). **E**. 3 dpf fish showing staining of a neuromast in the posterior lateral line (PLL). **F**. 3 dpf fish showing staining in multiple neuromasts in the trunk corresponding to the posterior lateral line (PLL) system. Scale bar: 50 μm.

In whole mount embryos, the expression patterns of Scn1ba_tv1 and Scn1ba_tv2 at 48 hpf and 72 hpf were found to be similar (Fig. 4). Zebrafish contain a set of mechanosensory organs, or neuromasts, that allow sensing of changes in water movement. These mechanosensory organs are divided into two categories: those in the anterior lateral line (ALL), e.g. localized in the head; and those in the posterior lateral line (PLL), e.g. localized in the trunk or tail [29]. Both anti-Scn1ba_tv1 and anti-Scn1ba_tv2 labeled neuromasts in the ALL and PLL (Fig. 4A–F) as early as 48 hpf. Additionally, both anti-Scn1ba_tv1 and anti-Scn1ba_tv2 labeled the olfactory pit (OP), another early developing sensory system (Fig. 4A and 4D). These results are consistent with the combined staining patterns for *scn1ba_tv1 *and *scn1ba_tv2 *antisense RNA observed in the *in situ *hybridization experiments, although mRNA expression was observed at earlier developmental stages than protein expression, suggesting a developmental delay between transcription and translation.

Fish that were 3, 5, 9, or 13 dpf were mounted in OCT and cryosectioned. Slices were stained with anti-Scn1ba_tv1 or anti-Scn1ba_tv2 antibodies as described in *Methods *and viewed with a fluorescent microscope. Sections were co-stained with anti-acetylated α-tubulin as a neuronal marker. By 3 dpf full expression was observed for both Scn1ba_tv1 and Scn1ba_tv2, as the pattern and intensity of staining did not change with ongoing development for either antibody. Staining patterns shown in subsequent figures are representative pictures for fish ages 3 through 13 dpf and are not reflective of a time course of expression for either subunit.

**Figure 5:**
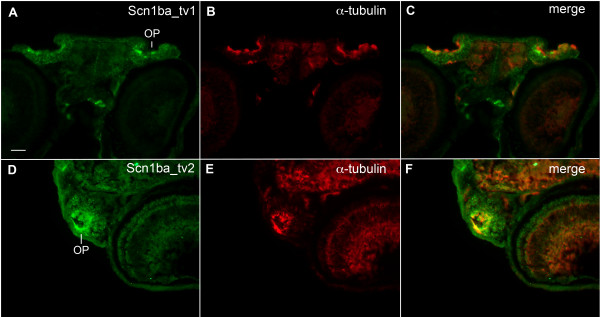
**Zebrafish anti-Scn1ba_tv1 and anti-Scn1ba_tv2 stain olfactory pits**. **A – C**: anti-Scn1ba_tv1 (green), anti-acetylated α-tubulin (red). **D – F**: anti-Scn1ba_tv2 (green), anti-acetylated α-tubulin (red). OP: olfactory pit. Scale bar: 50 μm.

**Figure 6:**
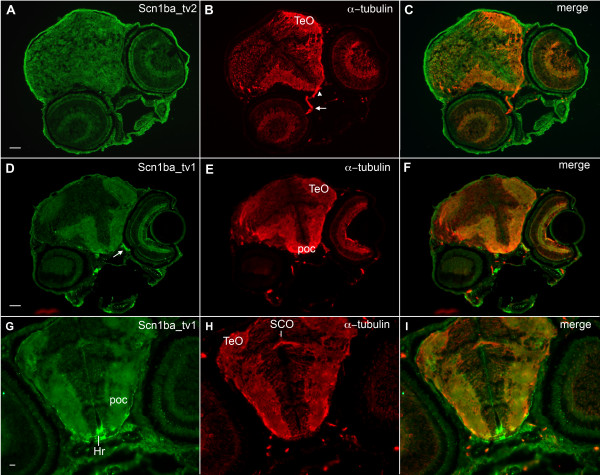
**Zebrafish Scn1ba_tv1 and Scn1ba_tv2 are expressed in brain**. **A – C**. Anti-Scn1ba_tv2 (green), anti-acetylated α-tubulin (red). Arrow: optic nerve. Arrowhead: optic chiasm. Anti-Scn1ba_tv2 does not stain the optic nerve or optic chiasm. Scale bar: 20 μm. **D – F**. Anti-Scn1ba_tv1 (green), anti-acetylated α-tubulin (red). Scn1ba_tv1 staining appears in the optic tectum (TeO), post optic commissure (poc), and optic nerve (arrow). **G – I**. Anti-Scn1ba_tv1 (green), anti-acetylated α-tubulin (red). Scn1ba_tv1 staining appears in the poc and TeO as well as in the rostral hypothalamus (Hr), but is absent in the subcommisural organ (SCO).

In agreement with antibody staining in whole mount embryos (Fig. 5), sectioned fish exhibited staining at olfactory pits (OP, Fig. 5) and this was observed for both Scn1ba_tv1 (Fig. 5A) and Scn1ba_tv2 (Fig. 5D). Dorsal views showed robust staining of both anti-Scn1ba_tv1 and anti-Scn1ba_tv2 that coincided with anti-acetylated α-tubulin (Fig. 5B and 5E). Examination of whole brain sections (Fig. 6) revealed anti-Scn1ba_tv1 staining in the optic nerve (Fig. 6D, arrow), tectum opticum (TeO), and post optic commissure (poc) (Fig. 6D–F). Anti-Scn1ba_tv1 stained rostral hypothalamus (Hr, Fig. 6G) independently of anti-acetylated α-tubulin but was absent from the subcommissural organ (SCO), where acetylated α-tubulin was strongly expressed (Fig. 6H). In contrast, there appeared to be generalized Scn1ba_tv2 staining that was diffuse throughout many brain regions (Fig. 6A–C). Most interestingly, anti-Scn1ba_tv2 staining was not detected in optic nerve (Fig. 6B, arrow) and optic chiasm (Fig. 6B, arrowhead).

**Figure 7:**
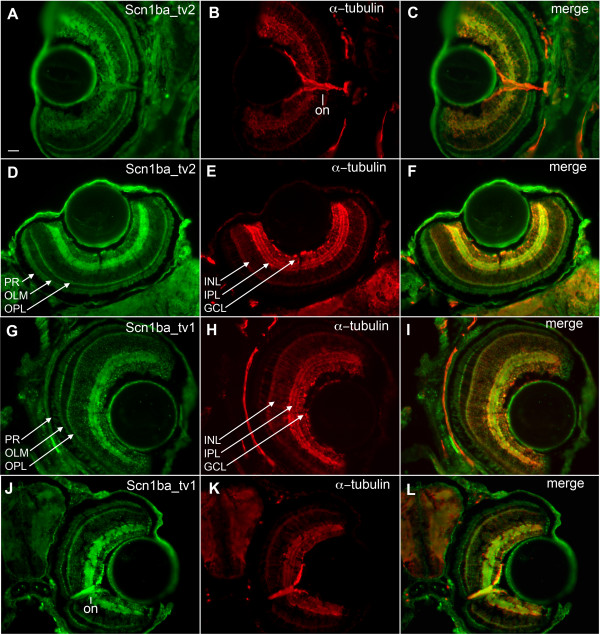
**Retinal patterning of Scn1ba_tv1 and Scn1ba_tv2**. **A – F**: anti-Scn1ba_tv2 (green), anti-acetylated α-tubulin (red). **G – L**: anti-Scn1ba_tv1 (green), anti-acetylated α-tubulin (red). Anti-Scn1ba_tv2 stains the layers of the retina, including the ganglion cell layer (GCL), inner plexiform layer (IPL), outer plexiform layer (OPL), outer limiting membrane (OLM), and photoreceptor cell layer (PR). Staining appears to be absent in the inner nuclear layer (INL) and in the optic nerve (on). Anti-Scn1ba_tv1 stains all the layers of the retina including the inner nuclear layer, where it shows robust staining. In contrast to anti-Scn1ba_tv2, anti-Scn1ba_tv1 labels optic nerve. Scale bar: 50 μm.

β subunit protein expression was examined in greater detail in the retina and optic nerve (Fig. 7). Anti-Scn1ba_tv2 staining was absent or weak in the optic nerve, indicated by the red staining in the merged image of optic nerve in Fig. 6A–B (arrow) and Fig. 7A (on). When we examined retinal patterning we found that anti-Scn1ba_tv2 stained throughout the layers of the retina with the strongest staining at the inner plexiform layer (IPL), outer plexiform layer (OPL), and outer limiting membrane (OLM) (Fig. 6D). Staining was weaker in the ganglion cell layer (GCL) and photoreceptor layer (PR), and weakest or absent in the inner nuclear layer (INL). In the INL, anti-acetylated α-tubulin appeared to label axonal tracts that were not labeled by anti-Scn1ba_tv2 (Fig. 7E). Labeling with anti-Scn1ba_tv1 demonstrated similar retinal patterning as Scn1ba_tv2 (Fig 7G–L). Zebrafish Scn1ba_tv1 is expressed in the IPL, OPL, and OLM. Similar to anti-Scn1ba_tv2, staining for anti-Scn1ba_tv1 was weaker in the GCL, PR, and INL (Fig. 7G). Most interestingly, Scn1ba_tv1 appeared to be robustly expressed in optic nerve (Fig. 7J), in contrast to Scn1ba_tv2, resulting in a yellow signal at optic nerve in the merged image (Fig. 7L).

**Figure 8:**
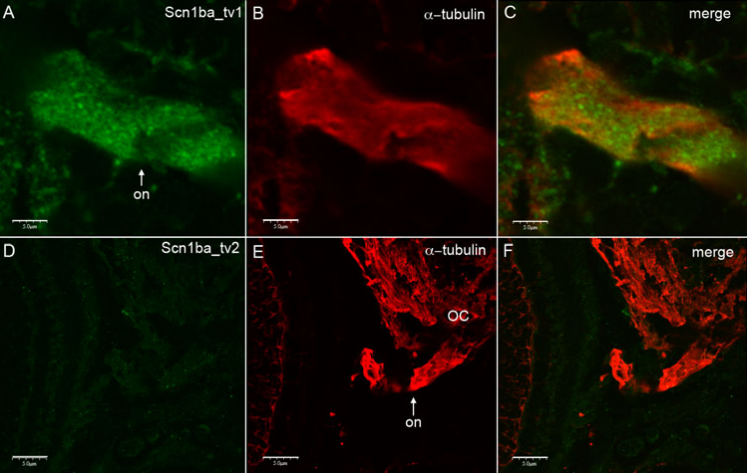
**Zebrafish Scn1ba_tv1 but not Scn1ba_tv2 is expressed in optic nerve**. Sections generated from 13 dpf zebrafish were stained with anti-Scn1ba_tv1 or anti-Scn1ba_tv2 and anti-acetylated α-tubulin. **A – C**: Anti Scn1ba_tv1 (green), anti-acetylated α-tubulin (red). **D – F**: Anti-Scn1ba_tv2 (green), anti-acetylated α-tubulin (red). Images were viewed with an Olympus FluoView 500 confocal microscope at 100× magnification with 5× additional zoom. Scale bar: 50 μm.

To further investigate the differential localization of Scn1ba_tv1 and Scn1ba_tv2 subunits in optic nerve, we analyzed cryosections generated from 13 dpf zebrafish by confocal microscopy (Fig. 8). Zebrafish optic nerves are surrounded with compact myelin at 7 dpf [30,31]. Thus, at this time point (13 dpf) we expected it might be possible to visualize staining at nodes of Ranvier if β1 subunits were indeed expressed in these specialized subcellular domains. Fig. 8A–C demonstrates the expression of Scn1ba_tv1 at optic nerve. Punctate staining suggests localization of this protein at nodes of Ranvier, consistent with our previous observations in mice [10]. In contrast to anti-Scn1ba_tv1, the anti-Scn1ba_tv2 antibody did not produce detectable staining in optic nerve (Fig. 8D–F). In order to further determine if Scn1ba_tv1 stained at nodes of Ranvier we dissected adult nerves and examined them at higher resolution in combination with antibodies for nodal and paranodal markers. Anti-Scn1ba_tv1 staining remained punctate and did overlap with nodes in some areas; however at this resolution we also observed significant background staining that made it impossible to confirm nodal staining (data not shown). Thus, with our current reagents we are unable to confirm that anti-Scn1ba_tv1 stains at nodes, although the punctate pattern is suggestive of nodal staining.

**Figure 9:**
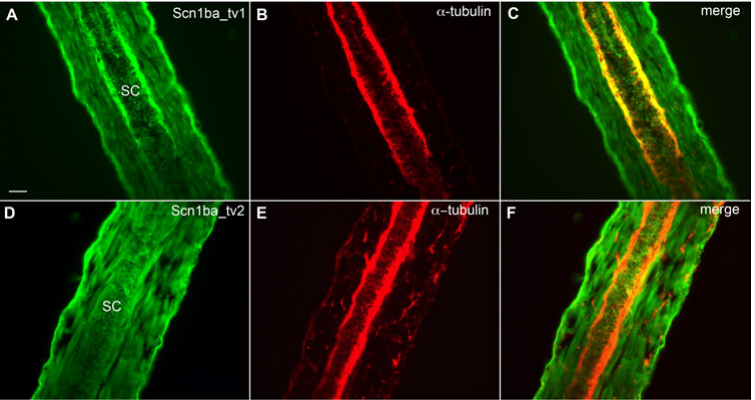
**Zebrafish Scn1ba_tv1 and Scn1ba_tv2 are differentially expressed in the spinal cord**. **A – C**: Anti-Scn1ba_tv1 (green), anti-acetylated α-tubulin (red). **D – F**: Anti-Scn1ba_tv2 (green), anti-acetylated α-tubulin (red). SC: spinal cord. Scale bar: 50 μm.

Next, we examined the localization of Scn1ba_tv1 and Scn1ba_tv2 in spinal cord using cryosectioned fish (Fig. 9). The results of our *in situ *hybridization experiments predicted expression of one or both subunits in this area. In longitudinal sections we observed strong anti-Scn1ba_tv1 staining in peripherally located fiber tracks that were positive for anti-acetylated α-tubulin (Fig. 9A–C). Anti-Scn1ba_tv1 staining was also observed in acetylated α-tubulin negative cells lining the central canal of the spinal cord (Fig. 9A–C). In contrast to Scn1ba_tv1, anti-Scn1ba_tv2 staining was observed in acetylated α-tubulin negative cells in the spinal cord but appeared to be absent from the peripheral acetylated α-tubulin positive fibers (Fig. 9D–F).

**Figure 10:**
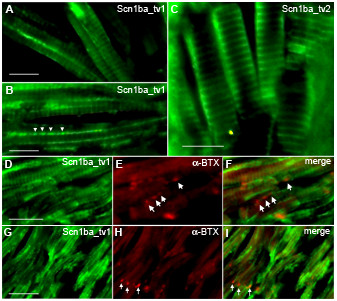
**Zebrafish Scn1ba_tv1 and Scn1ba_tv2 are expressed in skeletal muscle**. **A, B, D, E, F**: Anti-Scn1ba_tv1 β1 (green), α-bungarotoxin (BTX) (red). **C**. Anti-Scn1ba_tv2 (green). Labeling with anti-Scn1ba_tv1 produced two different staining patterns; staining at the t-tubules of striated muscle (A, D, and G), and punctate staining along the longitudinal edge of the muscle cells (arrowheads in B). Staining with anti-Scn1ba_tv2 labeled the t-tubule system and did not appear to label to muscle surface. Anti-Scn1ba_tv1 staining did not colocalize with α-BTX (D – F and H – I), suggesting that Scn1ba_tv1 is not expressed at neuromuscular junctions (arrows). Scale bar: 10 μm.

Zebrafish Scn1ba_tv1 and Scn1ba_tv2 are both expressed in striated skeletal muscle (Fig. 10), however, neither subunit was detected in cardiac muscle (data not shown). We observed two distinct patterns of skeletal muscle staining for anti-Scn1ba_tv1 (Fig. 10A,B, and 10D). This antibody labeled t-tubules, giving rise to the characteristic striated pattern of skeletal muscle (Fig. 10A), and stained in a punctate pattern along the longitudinal edges of the muscle cells (Fig. 10B, arrowheads). To investigate whether these puncta correspond to neuromuscular junctions, we stained with anti-Scn1ba_tv1 (Fig. 10D and 10G) and then co-stained with α-bungarotoxin (BTX) to mark neuromuscular junctions (arrowheads Fig. 9E and 9H); however, these two staining patterns did not overlap (Fig. 10F and 10I). Staining along the edges of the muscle is consistent with the localization of Na^+ ^channels at the muscle surface [32]. Anti-Scn1ba_tv2 staining (Fig. 10C) was also localized to the t-tubule system in skeletal muscle. Unlike anti-Scn1ba_tv1, however, punctate staining was not observed along the muscle edge, possibly revealing another tissue in which Scn1ba_tv1 and Scn1ba_tv2 are differentially localized. Similar to anti-Scn1ba_tv1, anti-Scn1ba_tv2 staining did not coincide with that of BTX (data not shown).

To summarize our immunohistochemical results, Scn1ba_tv1 and Scn1ba_tv2 are differentially localized in some tissues but not in others. Most notably, Scn1ba_tv1 is strongly expressed in optic nerve where punctate staining is suggestive of clustering at nodes of Ranvier, similar to mammalian β1 subunits [10], although we were not able to confirm this at high resolution. In contrast, we found no evidence for expression of Scn1ba_tv2 in optic nerve. Zebrafish Scn1ba_tv1 is also localized in peripheral, acetylated α-tubulin positive fiber tracts of the spinal cord and at the surface of skeletal muscle myocytes. No staining for anti-Scn1ba_tv2 was observed in these areas. Thus, Scn1ba_tv1 and Scn1ba_tv2 are differentially localized in some tissues *in vivo*, including brain, optic nerve, spinal cord, and skeletal muscle.

Clustering of Na^+ ^channels at mammalian axon initial segments and nodes of Ranvier is dependent on the expression of ankyrin and key L1 family cell adhesion molecules [33-36]. Previous results from our group have shown that mammalian β1-ankyrin association *in vitro *is dependent on the presence of a non-phosphorylated tyrosine residue in the β1 C-terminal domain [28]. Similar to β1, tyrosine phosphorylation of the intracellular domain of L1 family cell adhesion molecules, such as neurofascin, at the FIGQY motif abolishes their ability to interact with ankyrin, establishing specialized ankyrin-dependent and ankyrin-independent microdomains in neurons [37-40]. Non-phosphorylated neurofascin interacts with ankyrin_G _at nodes of Ranvier while tyrosine-phosphorylated L1 family cell adhesion molecules are found at other specialized sites of cell-cell contact such as paranodes of sciatic nerve, neuromuscular junctions, adherens junctions, and regions of neuronal migration and axon extension [37,41]. The FIGQY/H mutation in human L1 results in clinical disease, demonstrating that this tyrosine residue is critical for normal development of the nervous system [42-46]. We propose that β1 polypeptides containing a non-phosphorylated C-terminal tyrosine residue are localized with ankyrin_G _at nodes of Ranvier in myelinated axons. Tyrosine-phosphorylated β1 or β1-like subunits containing an alternate C-terminus, e.g. Scn1ba_tv2, are proposed to be localized to non-ankyrin-dependent domains where they are available to interact with other structural and signaling molecules, including different Na^+ ^channel α subunits. We have demonstrated that mammalian β1 retains its ability to associate with α subunits, but loses its ability to modulate Na^+ ^currents, when tyrosine-181 is mutated to glutamate to mimic phosphorylation [28]. Thus, we propose that differential localization of Scn1ba_tv1 and Scn1ba_tv2 in zebrafish may result in differential Na^+ ^current modulation and altered electrical excitability, depending on the specific association of α and β1-like subunits in different neuronal subpopulations. We have also shown that mammalian β1 promotes neurite outgrowth as a result of β1-β1 homophilic cell adhesion [9]. Our results suggest that extracellular β1-mediated homophilic adhesion activates an intracellular signal transduction cascade in the neuron. While the extracellular domains of Scn1ba_tv1 and Scn1ba_tv2 are identical, their intracellular domains are significantly different. Thus, while both β1-like subunits may function similarly in homophilic adhesion, their subsequent activation of intracellular signaling cascades is likely to be different and reflected in the resultant neuronal response. For example, we have shown that while β1 promotes neurite extension in cerebellar granule neurons, β2, which lacks an intracellular tyrosine residue, inhibits neurite extension [9]. It is possible that Scn1ba_tv1, which contains an intracellular tyrosine, promotes neurite outgrowth through a similar mechanism to β1, while Scn1ba_tv2, which does not contain an intracellular tyrosine, inhibits neurite outgrowth, similar to β2.

Interestingly, zebrafish neurons express two L1-related genes, *L1.1 *and *L1.2*, that have different C-terminal domains. The C-terminus of *L1.1 *is similar to mammalian L1 and contains the FIGQY motif. In contrast, and similar to Scn1ba_tv2, *L1.2 *contains a different C-terminal domain that does not include the FIGQY motif [47]. While *L1.1 *and *L1.2 *are encoded by separate genes in zebrafish and the immunohistochemical localization of these proteins has not yet been reported, the differences in their C-terminal domains are striking similar to the situation described in this study for Scn1ba_tv1 and Scn1ba_tv2 splice variants.

### Zebrafish scn1ba_tv1 and scn1ba_tv2 modulate Na^+ ^currents expressed by scn8aa

**Figure 11:**
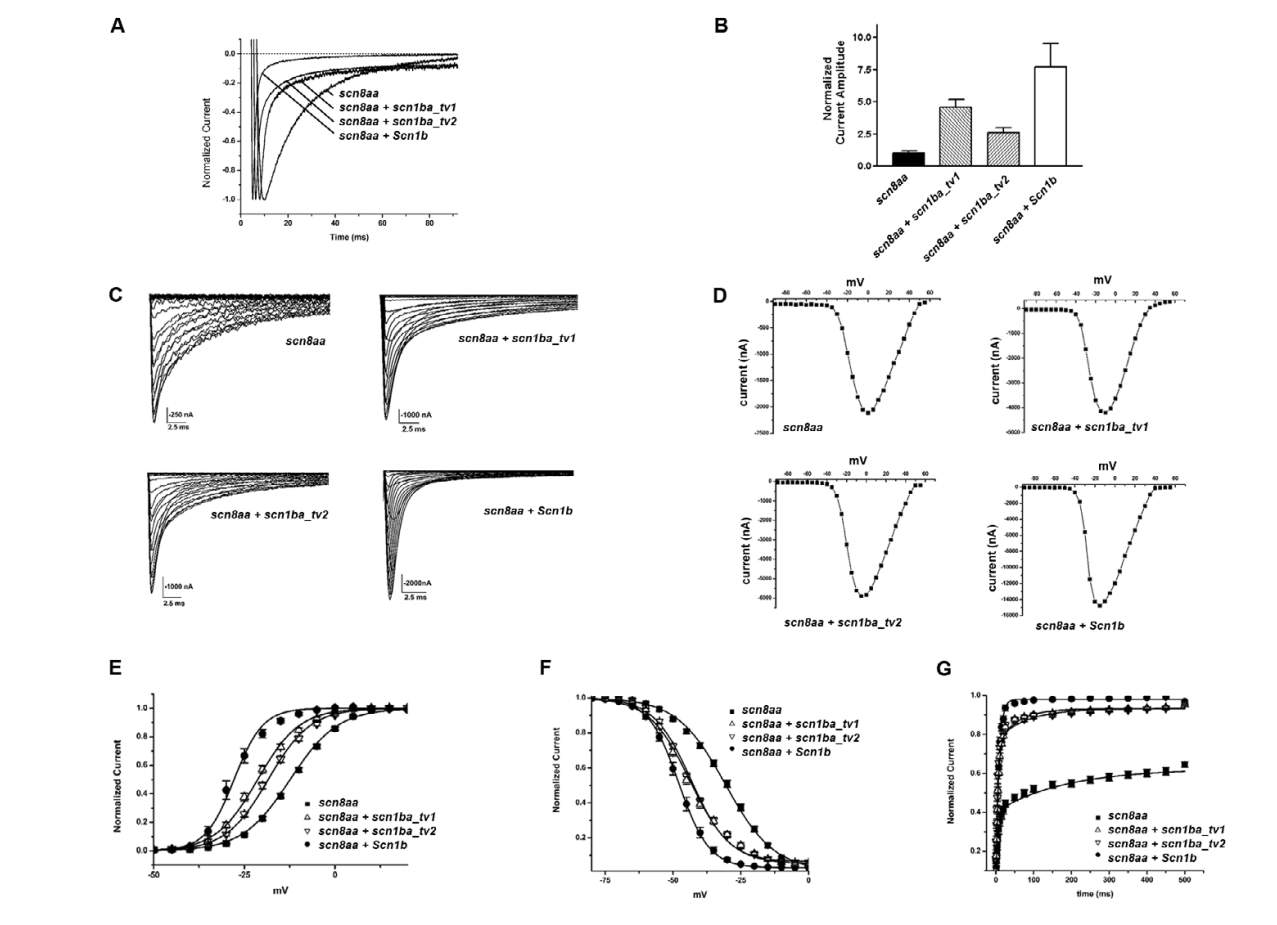
**Zebrafish β1 subunits modulate *scn8aa***. **A**. Normalized Current Traces. Effects of rat and zebrafish β subunits on current time course. Each trace shows the mean current elicited by depolarization to 0 mV from a holding potential of -80 mV in oocytes injected with the indicated combinations of α and β subunits. **B**. Current Density. Coexpression of *scn1ba_tv1*, *scn1ba_tv2*, or *Scn1b *cRNA with *scn8aa *cRNA results in increased current amplitude compared to α alone. Individual peak current amplitudes for each condition were measured and normalized to the mean current amplitude of *scn8aa *for each experiment to account for variability between different oocyte preparations. **C**. Representative Na^+ ^current traces for *scn8aa *alone (upper left), *scn8aa *plus *scn1ba_tv1 *(upper right), *scn8aa *plus *scn1ba_tv2 *(lower left), and *scn8aa *plus *Scn1b *(lower right) **D**. Current-voltage relationships for the families of Na^+ ^currents shown in panel C. **E**. Voltage dependence of activation. Coexpression of *scn8aa *with *Scn1b *(●), *scn1ba_tv1 *(△), or *scn1ba_tv2 *(▽) results in hyperpolarizing shifts in the voltage dependence of activation compared to the expression of *scn8aa *alone (■). Coexpression of *scn8aa *with *scn1ba_tv1 *results in a significantly greater hyperpolarizing shift than coexpression with *scn1ba_tv2*. **F**. Voltage dependence of inactivation. Coexpression of *scn8aa *with *Scn1b*, *scn1ba_tv1*, or *scn1ba_tv2 *resulted in hyperpolarizing shifts in the voltage dependence of inactivation compared to *scna8a *alone. The effects of *scn1ba_tv1 *and *scn1ba_tv2 *on the voltage-dependence of inactivation are indistinguishable from each other. **G**. Zebrafish β subunits speed recovery from inactivation. Coexpression of *scn8aa *with *Scn1b *(●), *scn1ba_tv1 *(△), or *scn1ba_tv2 *(▽) results in a dramatic increase in the rate of recovery from inactivation compared with *scn8aa *alone (■). Zebrafish *scn8aa *(■) expressed alone has a very slow rate of recovery, and a full recovery was never achieved during the duration of the experiment.

To investigate the effects of zebrafish β1 subunits on Na^+ ^channel expression and function, we injected *Xenopus *oocytes with *in vitro *transcribed cRNAs encoding either *scn1ba_tv1 *or *scn1ba_tv2 *together with cRNA encoding *scn8aa *(Na_v_1.6a) and examined the properties of the expressed Na^+ ^currents with two electrode voltage clamp recording. cRNA encoding rat *Scn1b *was coinjected with *scn8aa *for comparison. These data are the first report of functional expression of any zebrafish Na^+ ^channel α or β subunit. Co-expression of *scn8aa *with rat or zebrafish β1 subunits resulted in predicted shifts in channel gating mode from slow to fast (Fig. 11A and 11C), increases in current amplitude (Fig. 11B, C, and 11D), hyperpolarizing shifts in the voltage dependence of channel activation and inactivation (Fig. 11E and 11F), and increases in the rate of channel recovery from inactivation (Fig. 11G), effects that are characteristic of mammalian β1 subunits [3]. However, and as described below, while these effects were qualitatively similar between the rat and zebrafish β1 subunits, there were important quantitative differences in the extent of channel modulation.

To examine the effects of β1 subunits on the kinetics of Na^+ ^current activation and inactivation, we recorded macroscopic Na^+ ^currents. In response to depolarization, Na^+ ^currents expressed by all combinations of *scn8aa *± β1 subunits activated rapidly, although there was a slight speeding of the rate of current activation in the presence of *Scn1b*, *scn1ba_tv1*, or *scn1ba_tv2 *compared to *scn8aa *alone (Fig. 11A), as shown previously for *Scn1b *[48]. Shown in Fig. 11C are representative families of Na^+ ^current traces for the combinations of α and β1 subunits shown in panel A. Panel D shows representative current-voltage relationships for these α-β1 subunit combinations. In contrast to activation, the various combinations of *scn8aa *and β1 subunits exhibited very different time courses of current inactivation (Fig. 11A and 11C). In oocytes expressing *scn8aa *alone, the inactivation time course had two distinct components, a fast phase and a prominent slow phase that accounted for greater than 50% of the current, reflecting subpopulations of channels in fast and slow gating modes, respectively [49-51]. As shown previously for *Scn1b *[3,48,52], coexpression of *scn1ba_tv1 *or *scn1ba_tv2 *with *scn8aa *accelerated the inactivation time course by shifting the majority of channels to the fast gating mode. In contrast to coexpression of *Scn1b*, however, neither zebrafish β1 subunit fully shifted the population of channels to the fast gating mode, leaving a significant proportion of channels in the slow gating mode. Over the time course of the experiment, currents expressed by *scn8aa *alone or *scn8aa *coexpressed with *Scn1b *inactivated fully. In contrast, currents expressed by *scn8aa *and *scn1ba_tv1 *or *scna8aa *and *scn1ba_tv2 *did not inactivate completely, leaving a small fraction of the current that was non-inactivating.

Coexpression of either of the zebrafish β1 subunits with *scn8aa *increased Na^+ ^current amplitude compared to *scn8aa *alone, as shown previously for mammalian α and β1 subunits [3]. Fig. 11B presents mean peak current amplitudes recorded following injection of various combinations of *scn8aa *± β1 subunits. For each experiment, individual current amplitudes measured for each condition were normalized to the mean current measured in response to injection of *scn8aa *alone. Coinjection of *Scn1b *increased the Na^+ ^current amplitude by approximately 7.5-fold. Coinjection of the zebrafish β1 subunits also increased the current amplitude but to a lesser extent. Zebrafish *scn1ba_tv1 *increased the current amplitude approximately 5-fold while *scn1ba_tv2 *increased the current amplitude approximately 2.5-fold. Attempts at injecting higher concentrations of zebrafish β1 subunit cRNAs were unsuccessful, as this resulted in oocyte toxicity.

When we examined the voltage dependence of channel activation and inactivation we found that co-expression of *scn8aa *with *Scn1b *produced the expected hyperpolarizing shift, similar to that observed with mammalian Na^+ ^channel α and β1 cRNAs [3,53] (Fig. 11E and 11F). The half voltage of activation for *scn8aa *expressed alone was -11.84 ± 0.448 mV, and the half voltage of inactivation was -30.66 ± 0.740 mV. When co-expressed with *Scn1b*, the half voltage of activation was -27.85 ± 1.05, and the half voltage of inactivation was -47.89 ± 0.940 mV. These values represent significant -16.01 mV and -15.47 mV shifts, respectively (p < 0.001) and are similar to those reported for coexpression of mammalian *Scn8a *and *Scn1b *(for activation: V_1/2 _= -23 ± 1.9 mV; for inactivation: V_1/2 _= -51.1 ± 0.06 mV; [53]). Co-expression of *scn8aa *with *scn1ba_tv1 *or *scn1ba_tv2 *also produced leftward shifts in the voltage-dependence of activation and inactivation (Fig. 11E and 11F), however, while significant, these shifts were not as dramatic as those mediated by co-expression of *Scn1b*. The half voltage of activation for *scn8aa *plus *scn1ba_tv1 *was -21.33 ± 0.643 mV, a -9.48mV shift in comparison to *scn8aa *alone (p < 0.001). The half voltage of activation for *scn8aa *plus *scn1ba_tv2 *was -18.44 ± 0.589 mV, a -6.60 mV change compared to *scn8aa *alone (p < 0.001). Zebrafish *scn1ba_tv1 *and *scn1ba_tv2 *were significantly different from each other in their ability to modulate the voltage dependence of channel activation, with *scn1ba_tv1 *shifting 2.88 mV further in the hyperpolarizing direction than *scn1ba_tv2 *(p < 0.01). Both differed from the extent of change in the voltage-dependence of activation mediated by *Scn1b *(p < 0.01). The half voltage of inactivation for *scn8aa *co-expressed with *scn1ba_tv1 *was -43.70 ± 0.764 mV, a shift of -13.05 mV compared to *scn8aa *alone (p < 0.001). For *scn8aa *plus *scn1ba_tv2*, the voltage dependence of inactivation was -43.33 ± 0.618 mV, representing a shift of -12.67 mV compared to *scn8aa *alone (p < 0.001). Zebrafish *scn1ba_tv1 *and *scn1ba_tv2 *were not significantly different from each other in their ability to modulate the voltage dependence of channel inactivation (p > 0.05), however both differed from the extent of change in the voltage-dependence of inactivation mediated by *Scn1b *(p < 0.01). Interestingly, and in contrast to *Scn1b*, neither zebrafish subunit resulted in complete channel inactivation, suggesting that the zebrafish subunits modulate Na^+ ^channels differently than β1 subunits expressed in higher vertebrates.

We next examined the recovery time course of *scn8aa *inactivated by a 100 msec-long conditioning pulse to 0 mV in the presence and absence of β subunits (Fig. 11G). We observed that, similar to *Scn1b*, *scn1ba_tv1 *and *scn1ba_tv2 *both increased the rate of recovery from channel inactivation compared to *scn8aa *alone. However, in contrast to *Scn1b*, which completely shifted the rate of recovery from inactivation to the fast mode (98.5%), both zebrafish subunits left a significant percentage of slowly recovering channels. As shown in Table 1, the recovery time course for *scn8aa *alone was best fit with two exponentials, showing that the majority of channels recovered slowly, or not at all, for the duration of the experiment. In contrast, the recovery time course for *scn8aa *coexpressed with *Scn1b *was best fit by a single exponential, indicating that the fast component of inactivation predominated. For *scn8aa *coexpressed with *scn1ba_tv1 *or *scn1ba_tv2*, two exponentials were required to fit the data. For *scn8aa *and *scn1ba_tv1*, 16% of channels recovered slowly; for *scn8aa *and *scn1ba_tv2*, this value was 19%, suggesting again that the zebrafish β subunits modulate Na^+ ^channels differently than *Scn1b*.

**Table 1:** Recovery from Inactivation.

	τ_fast_, msec	%	τ_slow_, msec	%	n
*scn8aa*	6.501 ± 0.875	21.6 ± 3.8	203.478 ± 25.37	39.97 ± 6.9	19
*scn8aa *+ *Scn1b*	8.45 ± 0.773	98.5 ± 3.6			16
*scn8a*a + *scn1ba_tv1*	5.23 ± 0.585	76.98 ± 7.7	74.26 ± 10.3	16.33 ± 7.2	23
*scn8aa *+ *scn1ba_tv2*	3.89 ± 0.396	73.1 ± 8.9	55.84 ± 7.26	19.11 ± 9.3	22

Mean time-course of recovery from inactivation for Na^+ ^currents expressed by the indicated combinations of α and β1 subunits in *Xenopus *oocytes. Recovery time-course was assessed as described in *Methods*.

A common element in the mammalian and zebrafish β1 subunits is conservation of the extracellular Ig domain. Human mutations in the *SCN1B *extracellular Ig loop region result in epilepsy [54-56], suggesting that this region is critical for proper β1 function *in vivo*. Na^+ ^current modulation in oocytes depends on the extracellular Ig domain of *Scn1b *and does not require the intracellular domain [17], even though this domain contributes to the strength of α-β1 interactions [6,17,57]. Zebrafish *scn1ba_tv1 *and *scn1ba_tv2 *modulate Na^+ ^currents expressed in oocytes differently than *Scn1b *and the Ig loop region of the zebrafish subunits contains regions of divergence from the mammalian sequence that may account for these functional differences. As shown in Fig. 1, the C, C", F, and G strands are the most different from *Scn1b*, with the most significant differences in the C" region. The C" β sheet is also a site of divergence between the Ig domains of mammalian *Scn1b *and *Scn3b *subunits [18], although there is no homology between *Scn3b *and the zebrafish subunits in this region. If the extracellular domains of *scn1ba_tv1 *and *scn1ba_tv2 *are folded similarly to that of mammalian myelin P_o_, as predicted for *Scn1b *and *Scn3b *[17,18] (Fig. 1A, lower panel), then the C" strand is predicted to lie in an accessible region facing away from the α subunit-interacting A/A' face where it may interact with other cell adhesion molecules in the Na^+ ^channel complex. In contrast, the G strand, another region of divergence between the zebrafish and mammalian β1 subunits, lies parallel to the A/A' face and thus may interact with α. Dissimilarities in this region may be responsible for functional differences between these β1 subunits in terms of current modulation. Most notably, a proline residue located just prior to the G strand (P-134) may change the conformation of this region, causing the zebrafish subunits to favor the fast gating mode less effectively than *Scn1b*, as previously suggested for *Scn3b*, that also contains proline residues in this region of the Ig domain [18]. Thus, our results, taken in the context of previous studies, add important new information to the understanding of β1 subunit structure-function relationships.

## Conclusion

In the present study we demonstrate the first cloning, localization, and functional expression of two Na^+ ^channel β1 orthologs in zebrafish, *scn1ba_tv1 *and *scn1ba_tv2*, which arise from alternative splicing of *scn1ba*. We also show, for the first time, the functional expression of a zebrafish Na^+ ^channel α subunit, *scn8aa*. The deduced amino acid sequences of *scn1ba_tv1 *and *scn1ba_tv2 *are identical except for their C-terminal domains. The C-terminus of *scn1ba_tv1 *contains a tyrosine residue similar to that shown previously to be critical for β1-ankyrin association and β1-mediated Na^+ ^channel modulation in mammals [7,8,28]. In contrast, *scn1ba_tv2 *contains a unique, species-specific C-terminal domain that does not contain a tyrosine residue. Immunohistochemical analysis shows that, while the expression patterns of Scn1ba_tv1 and Scn1ba_tv2 overlap in some areas of the brain, retina, spinal cord, and skeletal muscle, only Scn1ba_tv1 is expressed in optic nerve. Both *scn1ba *splice forms modulate Na^+ ^currents expressed by *scn8aa*, resulting in shifts in channel gating mode from slow to fast, increased current amplitude, negative shifts in the voltage dependence of current activation and inactivation, and increases in the rate of recovery from inactivation, similar to the functioning of mammalian β1 subunits. In contrast to mammalian β1, however, neither zebrafish subunit produces a complete shift to the fast gating mode and neither subunit produces complete channel inactivation or recovery from inactivation.

Na^+ ^channel β1 subunits are multi-functional proteins that participate in multiple signaling pathways on time scales that range from msec (for modulation of Na^+ ^current) to hours (for stimulation of neurite outgrowth) [1]. We have shown previously, using gene-targeting strategies in mice, that β1 expression is critical for electrical excitability *in vivo *[10]. However, the severe neurological phenotype of *Scn1b *null mice has made a detailed analysis of β1 function *in vivo *quite challenging. With the cloning and functional expression of the zebrafish β1 ortholog, *scn1ba*, we are now poised to study the roles of the splice variants encoded by this gene in the development and maintenance of electrical excitability *in vivo *using a system that is more amenable to rapid genetic manipulation and analysis.

## Methods

### Animals

Zebrafish (*D. rerio*) were obtained from Doctor's Foster and Smith (Rhinelander, Wisconsin) and maintained at 28.5°C according to established procedures [11]. All animal protocols were approved by the University of Michigan Committee on Use and Care of Animals.

### Cloning of zebrafish scn1ba_tv1 and scn1ba_tv2

The translated Sanger zebrafish sequencing project database [58] was searched for homology to peptide sequences corresponding to rat Scn1b protein (GenBank AAH94523). To avoid incorporating sequencing errors into the data, several expressed sequence tags (ESTs) with similar sequences were aligned and amplification primers were designed to highly conserved regions. The oligonucleotides, CVEV (5'-GTGTGGAGGTCGACTCTG-3') and ASAT (5'-GTCCACCGTGGCGGAGGC-3'), forward and reverse primers, respectively, were used to amplify a short segment of a β1-like cDNA by polymerase chain reaction (PCR) from a zebrafish retina library (obtained from Dr. John Kuwada). The forward primer, CVEV, was then used in a 3' rapid amplification of cDNA ends (RACE) reaction following the manufacturer's instructions (Invitrogen, Carlsbad, CA). A nested primer, DTEA (5'-GACACAGAGGCAGTGGCGG-3') was used for a second round of amplification, resulting in the generation of two products which were later confirmed to be zebrafish β1 mRNA splice variants, "*scn1ba_tv1*" and "*scn1ba_tv2*", as described in *Results*. The 5' GeneRacer kit (Invitrogen) was used to amplify 5' untranslated regions, following the manufacturer's instructions, and including the gene specific primer, DTEAlong (5'-GCCTCTGTGTCAGAGTCGACCTCCA-3'). 2 μl of betaine were added to this reaction to aid in the amplification of identified GC rich sequences.

The identified cDNA clones were determined to be splice variants of a single gene by performing an independent RT-PCR reaction from RNA isolated from whole adult zebrafish using the Titan One Tube RT-PCR kit (Roche, Indianapolis, IN) and the oligonucleotides SKVM (5'-TCTGTGAAGATGTCTGCA-3') and SLKP (5'-AGCTTTTGGCTTGAGGCT-3'), as forward and reverse primers, respectively. Note: the sequences of *scn1ba_tv1 *and *scn1ba_tv2 *were recently reported in GenBank by another group (Accession numbers: DQ489725 and DQ489722, respectively).

### In Situ Hybridization

*In situ *hybridization was performed as previously described [11]. Briefly, zebrafish embryos at 24, 48, and 72 hpf were fixed overnight with 4% paraformaldehyde in PBS. Embryos were then dehydrated with ascending concentrations of methanol and stored at -20°C overnight before rehydration with descending concentrations of methanol and treatment with Proteinase K to increase permeability. Sense and antisense cRNA probes were generated using the DIG RNA labeling kit (Roche), following the manufacturer's instructions. Probe hybridization was performed overnight at 55°C and detected using an anti-DIG antibody conjugated to alkaline phosphatase, resulting in color development when reacted with nitroblue-tetrazolium-chloride/5-bromo-4-chloro-indolyl-phosphate. Images were collected using a Zeiss Axiophot fluorescent microscope and analyzed with Adobe Photoshop.

### Antibodies

Polyclonal antibodies to Scn1ba_tv1 and Scn1ba_tv2 subunit peptide sequences were generated by Affinity Bioreagents (Golden, CO) as fee-for-service. The peptide sequences of the antigens were as follows: Scn1ba_tv1: SESKDNCAGVQVAE, Scn1ba_tv2: EEALRESESKKSLKPK. β subunit antibodies were characterized by Western blot analysis of rat brain and zebrafish brain membranes and Chinese hamster lung 1610 cells transiently transfected with expression plasmids containing *scn1ba_tv1 *or *scn1ba_tv2 *cDNAs or with empty vector ("mock" transfection), as well as by immunohistochemical analysis of zebrafish retinal sections. Anti-acetylated α-tubulin (Sigma) was used as a positive control to stain neurons. These methods are described below.

### Western blot analysis

cDNAs encoding *scn1ba_tv1 *or *scn1ba_tv2 *were subcloned into pcDNA3.1 hygro (Invitrogen) and used to transiently transfect Chinese hamster lung 1610 cells [4]. Cells plated in 25 mm tissue culture dishes were transfected with 8 μg of cDNA using the Fugene 6 reagent (Roche) according to manufacturer's instructions. Cells were harvested 48 h following transfection. Rat brain or zebrafish brain membranes were prepared as previously described [28]. Samples were solubilized in SDS-PAGE sample buffer containing 1% SDS and 500 mM β-mercaptoethanol and heated to 70°C for 10 min before loading. Protein samples were separated on 10% polyacrylamide SDS-PAGE gels and transferred to nitrocellulose membranes. Membranes were then probed with antibodies to Scn1ba_tv1 or Scn1ba_tv2 at a concentration of 1:500 or with antibodies that had been preadsorbed to their corresponding immunizing peptide, as indicated in the figure legends, to demonstrate specificity. Preadsorption was performed by incubation of the primary antibody for 1 h at room temperature with an equal volume of the corresponding peptide diluted to a concentration of 1 mg/ml in phosphate buffered saline (PBS). Blots were then probed with HRP-conjugated goat anti-rabbit secondary antibody and detected with West Dura chemiluminescent reagent (Pierce).

### Immunohistochemistry

Immunohistochemistry was performed as previously described for whole mount embryos [11]. Briefly, embryos were fixed overnight at 4°C in 4% paraformaldehyde in PBS. Embryos were blocked in 10% heat inactivated goat serum, 0.5 mg/ml bovine serum albumin in PBS-T (containing 0.1% Tween) and stained overnight with anti-Scn1ba_tv1 or anti-Scn1ba_tv2 diluted 1:500 and co-stained with anti-acetylated α-tubulin (Sigma) diluted 1:2000. β subunit staining was detected using Alexa 488-conjugated anti-rabbit IgG and acetylated α-tubulin staining was detected using Alexa 594-conjugated anti-mouse IgG. Images were collected using a Zeiss Axiophot-2 fluorescent microscope equipped with a Zeiss Axiocam CCD digital camera and Axio Vision software and analyzed using Adobe Photoshop.

Immunohistochemistry was also preformed on fish cryosections as previously described. Briefly, fish were fixed in 4% paraformaldehyde for 1 h at room temperature and then cryoprotected in 30% sucrose overnight at 4°C before mounting in optimal cutting temperature compound (OCT). Once placed in OCT, fish were rapidly frozen on dry ice and stored at -80°C or used for immediate slicing. 10 μm sections were produced and used for subsequent staining. Embryos were blocked in phosphate buffer (0.02 M NaH_2_PO_4_, 0.08 M Na_2_HPO_4_) containing 0.3% triton X-100, and 10% goat serum. Embryos were incubated overnight with anti-Scn1ba_tv1 or anti-Scn1ba_tv2 antibodies diluted 1:500 or with antibodies preadsorbed to an equal volume of corresponding antigenic peptide resuspended to a concentration of 1mg/ml in phosphate buffered saline [see Additional file 1]. Anti-acetylated α-tubulin (Sigma) was used as a positive control to stain neurons. β subunit staining was detected using Alexa 488-conjugated anti-rabbit IgG and acetylated α-tubulin staining was detected using Alexa 594-conjugated anti-mouse IgG. For detection of neuromuscular junctions, slices were incubated with α-bungarotoxin conjugated to Alexa 594 (Invitrogen) for 30 min at room temperature. Images were collected using a Zeiss Axiophot-2 fluorescent microscope equipped with a Zeiss Axiocam CCD digital camera and Axio Vision software and analyzed using Adobe Photoshop. Images of optic nerve staining were also collected using an Olympus FluoView 500 confocal microscope and analyzed using Adobe Photoshop.

### Construction of a zebrafish scn8aa expression plasmid

Two partial cDNA clones encoding *scn8aa*, according to the described nomenclature [14], were obtained from Dr. Chi-Wei Tsai [15]. Plasmid #3844 contained the sequence corresponding to nucleotides 1–4009 of *scn8aa *in pBluescript SK+. Plasmid #53 contained the sequence of nucleotides 3372–6814 of *scn8aa *and was also in pBluescript SK+. The GenBank accession number for the complete sequence is AF297658. Both plasmids were digested with BstB1 and Xho1. A 7 kb fragment from plasmid #3844 was gel purified and dephosphorylated for use as the vector. A 2.9 kb fragment from plasmid #53 was gel purified and inserted into #3844. The two clones were ligated with T4 DNA ligase (Roche) and transformed. Selected clones were then digested with EcoRI to determine orientation. Clone ZEBRAFISH1.6BX#4 was identified as containing the correct sequence and used for subsequent studies.

### Electrophysiology

For whole cell recording of Na^+ ^currents expressed in *Xenopus *oocytes, *scn8aa*, *scn1ba_tv1*, *scn1ba_tv2*, and rat *Scn1b *cRNAs were synthesized using the T3 (*scn8aa*), SP6 (*scn1ba_tv1 *or *scn1ba_tv2*), or T7 (*Scn1b*) mMessage mMachine kits according to the manufacture's instructions (Ambion, Austin, TX) from plasmids linearized with either Xho I for *scn8aa*, Sma I for *scn1ba_tv1 *and *scn1ba_tv2*, or Not I for *Scn1b*. The resultant cRNAs were resuspended in RNA resuspension buffer (5 mM Hepes, 0.1 mM EDTA, pH 7.5) and samples of each preparation were analyzed by agarose-formaldehyde gel electrophoresis. Total mRNA yields for each preparation were estimated by comparing the intensity of ethidium bromide stained bands on agarose gels with the intensities of bands corresponding to RNA standards of known concentration.

*Xenopus laevis *oocytes were harvested, defolliculated with collagenase and maintained as described [59]. Briefly, pieces of ovary were surgically removed from female *Xenopus *frogs (Xenopus I, Ann Arbor, MI) anesthetized with 3-aminobenzoic acid ethyl ester. Oocytes were separated and defolliculated by shaking in 1.5 mg/ml collagenase in OR2 (82.5 mM NaCl, 2 mM KCl, 1 mM MgCl_2_, 5 mM HEPES, pH 7.5). Healthy stage V-VI oocytes were selected and incubated overnight at 18°C in Barth's medium (88 mM NaCl, 1 mM KCl, 0.82 mM MgSO_4_, 0.33 mM Ca(NO_3_)_2_, 0.41 mM CaCl_2_, 2.4 mM NaHCO_3_, 10 mM HEPES, pH 7.4), supplemented with 50 μg/ml gentamycin. On the day following isolation, oocytes were microinjected with 50 nl of RNA. The concentration of injected cRNA ranged from 50–300 ng/μl. We used approximately 5-fold greater concentrations of *scn8aa *cRNA to *Scn1b *cRNA, and 20-fold greater concentrations of *scn8aa *cRNA to *scn1ba_tv1 *or *scn1ba_tv2 *cRNA.

After incubation at 18°C for 48 h, expression of Na^+ ^currents was examined at room temperature by two-electrode voltage clamp using a TEV-200A amplifier (Dagan Corporation, Minneapolis, MN, USA) [59]. Voltage pulses were applied and data recorded on an IBM PC using the Clampex data acquisition system (Axon Instruments, Foster City, CA). Residual linear currents were subtracted using the P/4 procedure [60]. Signals were low pass filtered at 2 kHz using internal voltage clamp circuitry and data sampled at 20 kHz. The bath was perfused continuously with Frog Ringer solution containing 115 mM NaCl, 2.5 mM KCl, 1.8 mM CaCl_2_, 10 mM HEPES at pH 7.2. Intracellular pipette solutions contained 3 M KCl.

Following break-in to the cell, a period of 5 min was allowed for peak current levels to stabilize before initiating the electrophysiological recording. The voltage dependence of channel activation was determined from peak currents recorded during 90 msec-long test pulses to potentials ranging from -100 mV to 55 mV in 5-mV increments from a holding potential of -80 mV. Conductance (G) was calculated from peak current amplitude (I) according to *G *= *I*/(*V*-*V*_*rev*_) where *V *is the test potential and *V*_*rev *_is the measured reversal potential. The voltage-dependence of channel inactivation was measured using a 90 msec-long prepulse to potentials ranging from -100 mV to 55 mV followed by a test pulse to 0 mV. Conductance-voltage curves and inactivation curves were fit with the Boltzmann relationship, *G *= 1/(1 + exp((V-V_1/2_)/*k*)) where V_1/2 _is the midpoint of the curve, and *k *is a slope factor. The time constant, τ, of current inactivation was obtained by applying the sum of either one or two exponentials to the decay phase of currents obtained during investigation of the voltage dependence of activation. To determine the time course of recovery from inactivation, Na^+ ^currents were inactivated with a 100 msec-long pulse to 0 mV, which was followed by a recovery prepulse of variable duration to -80 mV, and a subsequent test pulse to 0 mV to determine the fraction of recovered channels. Recovery data were fit with either a single or double exponential to determine the time constant(s) for recovery from inactivation. Statistical significance between groups was determined using one-way ANOVA followed by post hoc Tukey analysis. Differences were considered to be significant when p < 0.05. Electrophysiological data were analyzed with pCLAMP software (Axon Instruments, Foster City, CA) and plotted with Origin (Micrococal, Northampton, MA) or SigmaPlot (Jandel, San Rafael, CA).

## Abbreviations

ALL anterior lateral line

BAC bacterial artificial chromosome

BTX α-bungarotoxin

dpf days post-fertilization

EST expressed sequence tag

GCL ganglion cell layer

Hb hindbrain

hpf hours post-fertilization

INL inner nuclear layer

IPL inner plexiform layer

LG linkage group

Mb midbrain

OCT optimal temperature cutting compound

OLM outer limiting membrane

on optic nerve

OP olfactory placode

OPL outer plexiform layer

PLL posterior lateral line

PR photoreceptor layer

RB Rohan Beard

sc spinal cord

sm skeletal muscle

Tg trigeminal

UTR untranslated region

## Authors' contributions

AJF carried out the *scn1ba *molecular cloning studies, the electrophysiology studies, the immunohistochemical studies, characterized the antibodies, generated the sequence alignment, generated all of the figures, and helped to draft the manuscript. LSM trained AJF in the required electrophysiological techniques, participated in analysis of the electrophysiological data, and helped to draft the manuscript. CC assembled the *scn8aa *clone, verified it by DNA sequencing, and subcloned it into the oocyte expression vector. EAS performed the cell transfections and Western blots. LLI conceived the study, participated in its design and coordination, and drafted the manuscript. All authors read and approved the final manuscript.

## Supplementary Material

Additional file 1 Antibody characterization. Immunohistochemical analysis of anti-Scn1ba_tv1 (top panel) or anti-Scn1ba_tv2 (lower panel) antibody (green) staining following pre-adsorption to its corresponding antigenic peptide. Sections were co-stained with anti-acetylated α-tubulin (red). Merged panels on the right. Click here for file
