# Supplementary figures and images for: Cloning and expression of a zebrafish SCN1B ortholog and identification of a species-specific splice variant
Source: BMC Genomics. 2007 Jul 10;8:226. doi: 10.1186/1471-2164-8-226 (PMC1965480; doi:10.1186/1471-2164-8-226)

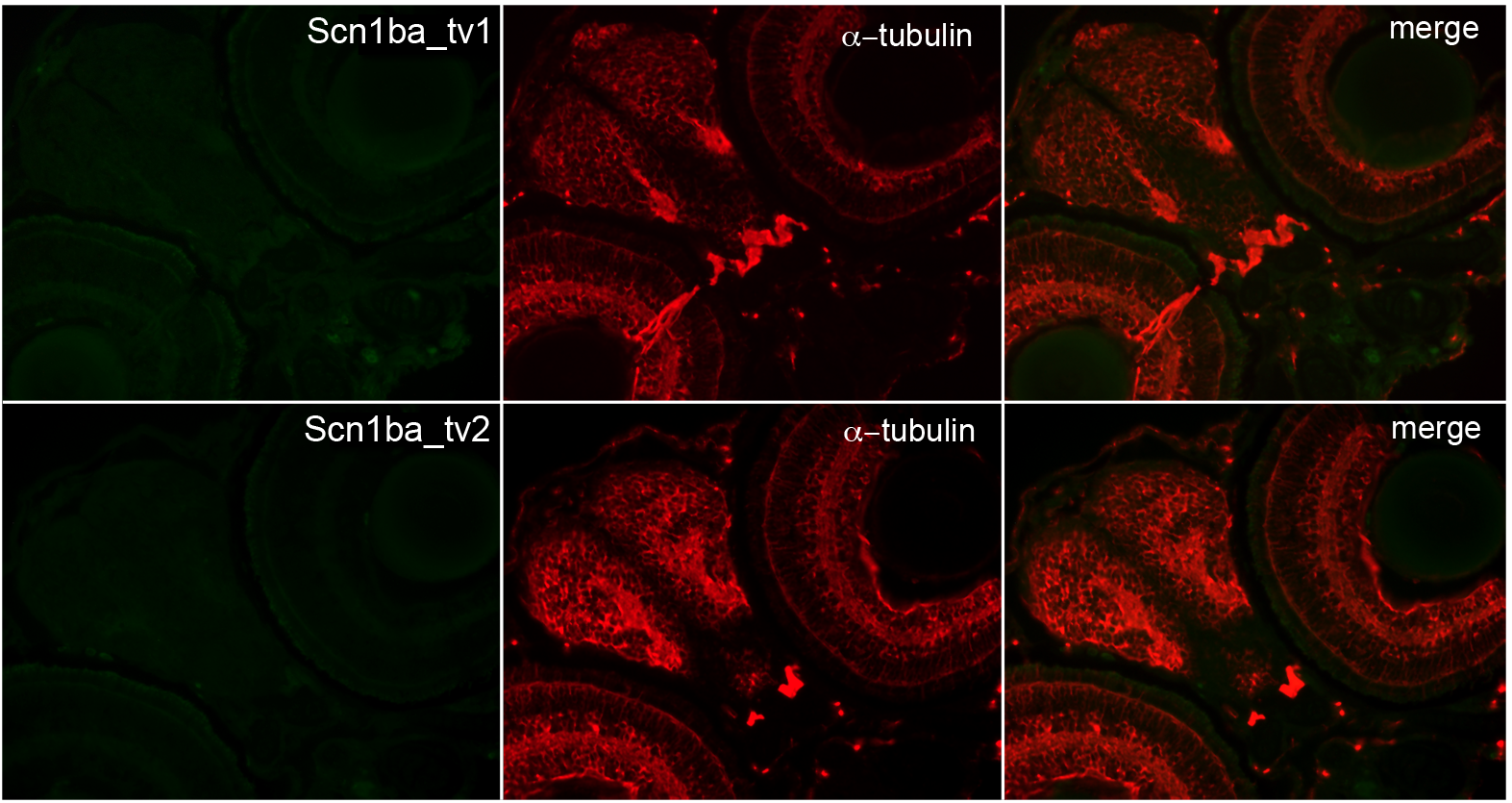

Supplement: Additional file 1 — Antibody characterization. Immunohistochemical analysis of anti-Scn1ba_tv1 (top panel) or anti-Scn1ba_tv2 (lower panel) antibody (green) staining following pre-adsorption to its corresponding antigenic peptide. Sections were co-stained with anti-acetylated α-tubulin (red). Merged panels on the right. [file 1471-2164-8-226-S1.tiff]
